# Tribological Performance of Short Fibers Reinforced Thermoplastic Polyurethane Composite Materials Under Water-Lubricated Condition

**DOI:** 10.3390/polym17010030

**Published:** 2024-12-26

**Authors:** Yicong Yu, Pan Jiang, Wei Yu, Zhiwei Guo

**Affiliations:** 1 School of Transportation and Logistics Engineering, Wuhan University of Technology, Wuhan 430063, China; yicongyu@whut.edu.cn; 2 State Key Laboratory of Maritime Technology and Safety, Wuhan University of Technology, Wuhan 430063, China; 3 Reliability Engineering Institute, National Engineering Research Center for Water Transportation Safety, Wuhan 430063, China; 4 Wuhan Branch of China Classification Society, Wuhan 430022, China; 3220245434@bit.edu.cn

**Keywords:** water-lubricated bearing, fiber-reinforced composites, thermoplastic polyurethane, tribological performance

## Abstract

The water-lubricated bearing plays a crucial role in the ship propulsion system, significantly impacting vessel safety. However, under the harsh working conditions of low-speed and heavy-load, the lubrication state of water-lubricated bearings is usually poor, leading to serious friction and wear. To improve the tribological performance of composites and reduce friction, three short fibers (ultra-high-molecular-weight polyethylene fibers, basalt fibers, and bamboo fibers) with the same mass fraction (5%) were added into the melted thermoplastic polyurethane (TPU). The tribological behavior of these three composites under different loads and rotation speeds was investigated using the CBZ-1 friction and wear tester. Through the comprehensive analysis of the friction coefficient, the wear mass loss, and the surface morphology, it was confirmed that the filled fiber positively affected the tribological performance of thermoplastic polyurethane materials. The experimental results indicated that basalt fiber significantly improved the tribological performance of TPU, and the friction coefficient of the sample was only 0.088 under the working conditions of 0.5 MPa and 250 r/min, which was 70.57% lower than that of pure TPU material. And in all the tests, the minimum wear of the basalt fiber-reinforced composite is only 0.4 mg, which is also the smallest of all the materials under all conditions, and a decrease of 98.69% compared to TPU. Under high loads, ultra-high-molecular-weight polyethylene fiber and bamboo fiber-reinforced composites have smoother surfaces and exhibit better tribological properties. This study provides an experimental foundation for tribological performance enhancement for environmentally friendly, water-lubricated bearing composites.

## 1. Introduction

It is necessary to protect the environment and resources that oil-lubricated bearings need to be replaced because they might consume massive mineral oil and precious metal resources. It has been a trend to replace oil-lubricated bearings with water-lubricated bearings in the marine field because they meet the requirements of green shipping [1]. However, the stern tube journal bearing of a ship operates under frequent start–stop operation due to ship steering [2], which makes the bearing bushing in the unstable stress environment. At the same time, the viscosity of water is very low, and it is difficult to form a stable lubricating water film under harsh conditions, resulting in poor carrying capacity [3]. These harsh operating environments will cause obvious friction and wear of water-lubricated bearings, which directly affects the service life of bearings and the safety of ships. Therefore, it is of great significance to study the friction and wear properties of bearing materials under water lubrication for improving the lubrication conditions of friction pairs.

Polymers possess properties such as low friction, good wear resistance, and corrosion resistance, making it desirable for stern tube bearing applications [4]. Common polymer substrates are ultra-high-molecular-weight polyethylene (UHMWPE) [5], polytetrafluoroethylene (PTFE) [6], nitrile butadiene rubber (NBR) [7], and other materials, which provide the necessary strength for the bearing. Among these polymer matrix alternatives, thermoplastic polyurethane (TPU) has been studied widely due to its characteristic structure of soft/hard segments, providing excellent and tunable mechanical properties [8].

**Figure 1:**
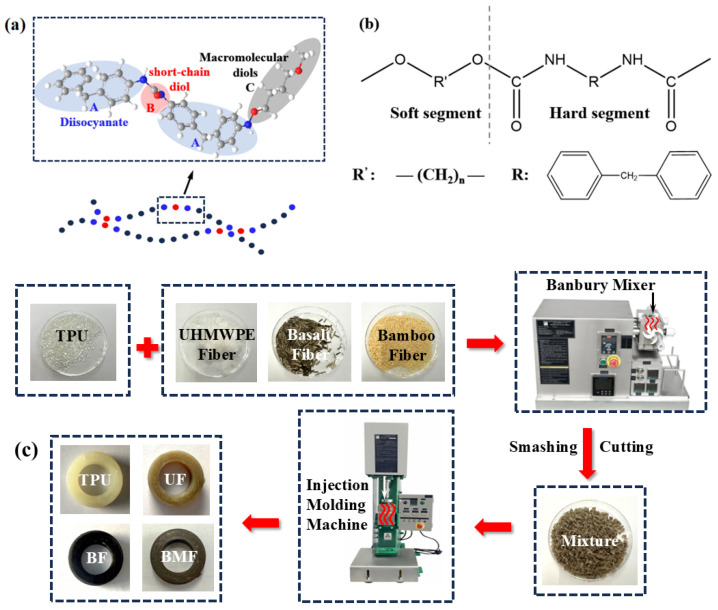
(**a**) Molecular chain of TPU, (**b**) structural formula of TPU, and (**c**) preparation process of composite material sample.

TPU is a multiblock copolymer whose molecular chain consists of a “hard segment” (HS) composed of diisocyanate and short-chain diol and a “soft segment” (SS) composed of macromolecular diols. Their molecular formula is shown in Figure 1b. The hard segment and soft segment are arranged alternately on the molecular chain [9], as shown in Figure 1a. SS exhibits strong mobility; the higher its content and the longer the chain segment, the better the elasticity and toughness of TPU. Conversely, when the content of HS is higher, the chain segment becomes less mobile, increasing the hardness and rigidity of TPU. The unique structure of TPU combines characteristics of both plastic and rubber. This dual nature enhances the tribological and mechanical performance of water-lubricated bearings made from TPU. Therefore, TPU is also widely used to improve the tribological properties of composites. The results show that TPU and polyamide (PA) composites exhibit good tribological properties under different sliding conditions. The toughness of PA6-TPU composites is significantly improved, and the friction coefficient stabilizes rapidly at low values, but the wear rate increases under high-load conditions [10]. In the biomedical field, biocompatible TPU composites containing titanium particles show excellent wear resistance. With the increase in titanium particle content, the hardness of the composite gradually increased, and the wear coefficient and wear rate reached the lowest at 2 wt%, but increased at 4 wt% due to the increase in volume loss [11]. The microfiber network of TPU and polytetrafluoroethylene (PTFE) composite was prepared by melting and mixing, and the friction coefficient was significantly reduced. It was found that the size of PTFE fiber is closely related to the shear stress, and the higher shear stress helps to form a thinner fiber, thereby improving the tribological properties of the composite [12].

In order to meet the demand for lightweight, high-strength, and durable materials in the high-tech field, high-performance fiber composites with various high-performance fibers as reinforcement materials have been rapidly developed. With the advancement of blending modification technology, new fiber-reinforced thermoplastic resin composites with new functions and high quality and low price are emerging one after another. UHMWPE fibers are known for their high strength-to-weight ratio, excellent abrasion resistance, and good impact resistance, which makes them ideal for enhancing the durability and mechanical strength of TPU composites [13]. Basalt fibers provide excellent thermal stability, high strength, and good chemical resistance. They contribute to the structural integrity and overall performance of TPU-based composites, especially in high-temperature applications [14]. Bamboo fibers are a sustainable and eco-friendly option that offers good mechanical properties, such as high tensile strength and flexibility. They also contribute to the biodegradability of the composite, making it a more environmentally conscious choice [15].

In this paper, three kinds of short fiber were selected as the reinforcing phase of the composites, and the TPU-based fiber-reinforced composites were prepared by injection molding. Under the condition of water lubrication, the CBZ-1 Marine shafting friction and wear testing machine was used to compare different fiber-reinforced composites (FRCs) under different working conditions, and the wear mechanism was analyzed by the surface morphology after the test. This study provides an experimental and theoretical basis for enhancing the tribological performance of environment-friendly, water-lubricated bearing composites.

## 2. Materials and Methodology

### 2.1. Materials

Neat thermoplastic polyurethane (Polyester TPU S172DL) was purchased from KOSLEN (Fujian, China) Co., Ltd. Ultra-high-molecular-weight polyethylene fibers were purchased from Sinopec Yizheng Chemical Fibre (Zhejiang, China) Co., Ltd. Basalt fibers were purchased from NCE Composites (Jiangsu, China) Co., Ltd. Bamboo fibers were purchased from Rui Mei bamboo technology (Jiangxi, China) Co., Ltd. Professional distilled water was purchased from Watsons (Beijing, China) Co., Ltd.

### 2.2. Specimen Preparation

In this study, all TPU-based samples were obtained by melt injection molding, which is convenient and cost-effective. First, three fibers and TPU of the same quality are added to the Banbury mixer, which is stirred at 180 °C for a period of time. The mixture is then crushed and cut into small particles, which are finally placed in an injection molding machine to obtain a test sample at a certain pressure and temperature. The preparation process is shown in Figure 1c.

Four types of samples were prepared as follows: pure thermoplastic polyurethane (TPU), TPU+5 wt% UHMPWPE fiber (named UF), TPU+5 wt% basalt fiber (named BF), and TPU+5 wt% bamboo fiber (named BMF). These three fibers were chosen as reinforcement phases because they mostly have good mechanical properties and a positive impact on the environment and are potential materials for water-lubricated bearings [13,14,15].

**Figure 2:**
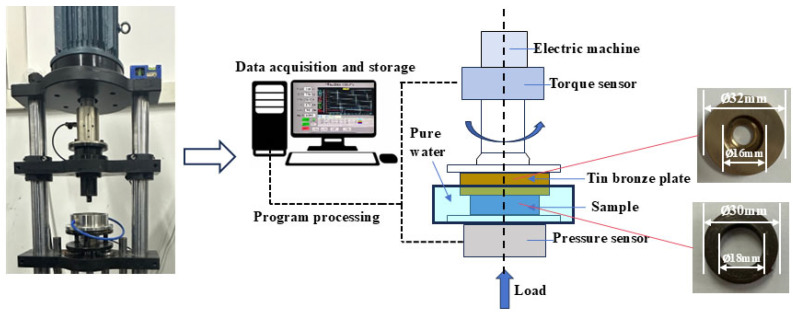
CBZ-1 friction and wear testing machine and schematic diagram.

The test simulated the real working environment of the ship stern shaft and bearing under the condition of water lubrication through the contact between the tin bronze plate and sample. The sample is made of a ring shape with an outer diameter of 30 mm, an inner diameter of 18 mm, and a height of 10 mm. In addition, QSn7-0.2 is a commonly used material for shaft bushings in ship shafting, so tin bronze of the same material was selected as the grinding part of FRC in the test [16]; the element composition is shown in Table 1. In order to ensure the fully stable contact of the friction pair in the tribological test, the surface size of the tin bronze plate is designed to be slightly larger than the sample surface size. Among them, the outer diameter of the tin bronze plate is 32 mm, the inner diameter is 16 mm, and the thickness is 6mm. The morphology and size of the friction pair are shown in Figure 2. The mechanical properties of TPU and tin bronze plates used in the test are shown in Table 2 and Table 3, respectively.

### 2.3. Materials Characterization Techniques

A universal material testing machine (Instron 5967, Co., Ltd., Boston, MA, USA) was used to measure the stress–strain curve of the material in a uniaxial tensile test according to the testing standard ISO 527-1 [17]. Shore hardness of samples was tested by a Shore hardness tester (HLX-AC, Adelberg Handpi Instrument Co., Ltd., Wenzhou, China). The surface morphology of the polymer disk was observed by scanning electron microscope (VEGA3, Tescan Co., Ltd., Brno, Czech Republic). Before scanning electron microscopy (SEM) measurements, all samples were gold-plated by sputtering. Laser scanning confocal microscope (VK-X2000, Keyence Ltd., Osaka, Japan) was used to observe the wear surface of the grinding pair.

### 2.4. Tribological Test

The CBZ-1 friction and wear testing machine (Haima Co., Ltd., Wuhan, China) was used to conduct a tribological performance test. The testing machine was composed of the host rotating system, data acquisition, and processing system. The physical drawing, working principle, and test details were shown in Figure 2.

The sample is fixed between the testing machine base and the tin bronze plate and is completely immersed in pure water. The tin-bronze grinding piece is driven by a motor to slide on the surface of the sample, and the experimental load and speed are adjusted by the control device of the friction and wear testing machine, so that the sample ring and the tin bronze plate are subjected to disc grinding tests. During the experiment, different sensors were used to collect the pressure, torque, and other parameters, and the friction coefficient was calculated in real time by the LabVIEW system in the computer. Calculate the real-time friction coefficient of the CBZ-1 friction and wear testing machine according to Equation (1) as follows:

(1) $$\mu =\frac{T}{rF}$$

where *μ* represents the friction coefficient; *T* stands for torque, N · m; *F* stands for load, N; *r* represents the sample rotation radius, m.

The test conditions were selected according to the U.S. Navy military standard MIL-DTL-17901C [18]. The speed of the test spindle is set to 50, 150, and 250 r/min to simulate the working condition of the ship at low speed and normal operation. Since the nominal pressure of the ship’s water-lubricated tail bearing is below 0.55 MPa [9], the load applied on the friction pair is set to 0.5 and 0.7 MPa to simulate the normal load and heavy load conditions, respectively, so as to explore the water lubrication performance of FRC under normal and harsh conditions. In order to ensure the reliability of the test results, the test is repeated three times under each friction and wear test condition to eliminate accidental errors.

Before the test, the surfaces of the tin bronze plate and composite material samples were polished with 240 mesh, 600 mesh, and 1000 mesh sandpaper on the polishing machine, respectively, so that the surface roughness of the friction pair was the same, about 0.3 µm. The polished material is rinsed with distilled water, placed in the oven for 12 h, and weighed. Each tin bronze plate and composite material sample is tested 3 times to calculate the average mass. In the test, when changing the material or changing the working condition, the tank was cleaned with distilled water, and the grinding was continued for 7200 s under each working condition. After the test, the tin bronze plate and composite material samples were washed again with distilled water, dried in the oven for 12 h, and weighed. Each tin bronze plate and composite material sample was recorded 3 times, and the average value was taken. By comparing the sample quality before and after the test, the wear data of the sample during the friction and wear test can be calculated. After the friction and wear test, the wear morphology of FRC material was observed by scanning electron microscope (SEM) and compared with that of pure TPU.

## 3. Results and Discussion

### 3.1. Water Absorption

Considering that there are organic fibers in the reinforced fibers added in this study, the addition of these fibers may cause large expansion in water, which may have an impact on the development of materials used in water-lubricated bearings [19]. Therefore, it is necessary to study the water absorption of the four different material samples in this test. Four samples with dimensions of 30 mm × 10 mm × 10 mm were soaked in distilled water in a 1 L beaker at 25 ° C. Vernier calipers were used to measure and record the average volume change of the sample under different soaking times. Table 4 shows the average volume changes in samples soaked in distilled water for different times.

According to the US military standard MIL-DTL-17901C (SH) [19], for water-lubricated bearings made of plastic material as a substrate, the change in volume of the sample made of this material after soaking in water for 7 days should be less than 5%, and if the volume increase is less than 1% and 2%, it is grade IV and grade V, respectively.

The test results show that the volume growth rate of BMF with the worst water absorption performance in this test is less than 1%, which is in line with military standards. After adding UHMWPE fiber and basalt fiber, the water absorption performance of TPU has been improved. BF has the best water absorption performance and can meet the requirements of grade IV standards and is beneficial to the safe navigation of ships as a water-lubricated bearing material.

### 3.2. Mechanical Properties

**Figure 3:**
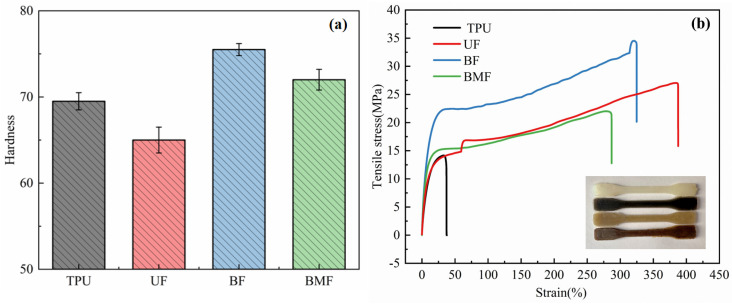
Mechanical properties of TPU− based samples: (**a**) hardness and (**b**) tensile stress−strain curve. The illustrations in (**b**) are dumbbell-shaped samples for tensile testing.

Mechanical properties are one of the most important parameters to determine the applicability of engineering materials [20]. Moreover, in the field of tribology, the mechanical properties of materials are also one of the important reasons for the difference in tribological properties. Therefore, the hardness (Shore D) and tensile properties of TPU composites were measured in this paper. Figure 3 shows the test results of hardness and tensile strength of four different materials.

Figure 3a shows that the hardness of BF and BMF is higher than that of TPU, but the hardness of UF is lower than that of TPU. This shows that the addition of basalt fiber and bamboo fiber to the TPU matrix has a positive effect on the hardness of the composite; however, the addition of UHWMPE fiber will reduce the hardness of the composite, which may be due to the phase separation phenomenon between TPU and UHWMPE. In addition, UHMWPE has excellent self-lubricating properties, which, in some cases, reduce the friction coefficient and hardness of the material, making the material more susceptible to deformation under external forces [21]. At the same time, the hardness of BF is relatively highest, not only because of the high hardness of the basalt fiber itself but also because the presence of the fiber improves the structural integrity of the material and reduces the generation and expansion of cracks; the overall performance of the composite material is improved [22].

The stress–strain curves of the four materials can be seen in Figure 3b. The elongation at break of the materials in descending order is UF (390%) > BF (325%) > BMF (290%) > TPU (50%). However, the tensile strength of the material is BF (35 MPa) > UF (27 MPa) > BMF (22 MPa) > TPU (14 MPa) from large to small. The elongation at break and tensile strength of UF, BF, and BMF are higher than those of TPU, which indicates that the addition of three kinds of short fibers to the TPU matrix will lead to the improvement in the ductility and tensile strength of the composite. Among them, UF has the highest elongation at break, while BF has the highest tensile strength, which indicates that UF is very flexible and can have huge deformation before fracture, but the stress it can withstand is less than BF. This causes the UF to begin to deform at lower stresses, but because the structure is able to absorb energy, the material does not break until it deforms [23]. For BMF, after adding bamboo fiber, the hardness, tensile strength, and elongation at break of TPU material were slightly improved, but the effect was much weaker than that of UHMWPE fiber and basalt fiber, which may be related to the strength of plant fiber itself.

### 3.3. Friction Coefficient Analysis

**Figure 4:**
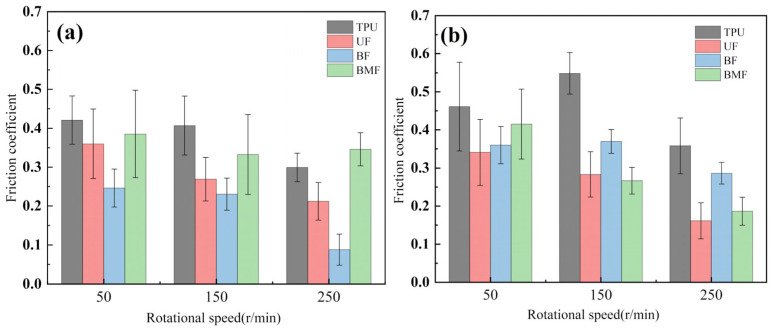
Friction coefficients of specimens under different loads: (**a**) 0.5 MPa and (**b**) 0.7 MPa.

Friction coefficient is one of the most important reference parameters that describes the tribological properties of material [24]. In order to better describe the tribological properties of materials, the numerical value, stability and variation trend of friction coefficients under different test conditions will be comprehensively considered in this section. In the test, the friction coefficients of different samples were recorded in real time to explore the tribological properties of short fiber-reinforced TPU materials under water lubrication, as shown in Table 5. In order to ensure the rigor of the test, it is necessary to consider the friction pair in the initial running-in stage of the test, and calculate the average friction coefficient of the sample under different loads starting from the friction coefficient after the 2000’s, as shown in Figure 4.

It can be seen from Figure 4 that the friction coefficient of pure TPU samples is the largest under most working conditions of this test, which proves that the addition of three staple fibers can improve the tribological properties of TPU composites to varying degrees. At 0.5 MPa pressure, the friction coefficient value of BF is the smallest, which is 70.57% lower than that of pure TPU. Interestingly, however, UF and BMF may show smaller friction coefficient values at 0.7 MPa pressure. This is related to the properties of the fiber itself and the interface bonding between the fiber and the TPU matrix. Under the pressure of 0.5 MPa, basalt fiber provides good support for the sample with its high hardness and stiffness, reduces the contact area between the friction pairs, and has good lubrication status, which helps to improve the wear resistance of the composite material and reduce the friction coefficient. UHMWPE fiber can also reduce the friction coefficient of composite materials, but the effect is not as significant as basalt fiber under relatively low load conditions. However, when the pressure is increased to 0.7 MPa, huge pressure and constant relative rotation of friction pairs will accumulate a large amount of friction heat on the surface of the sample. When the surface temperature of the TPU composite is close to or exceeds the glass transition temperature (Tg) of the hard segment (HS), the hard domain will be oriented into type III crystals [25], which is conducive to a longer extension of the soft segment (SS). It is easy to cause the deformation of TPU, which affects the stick-slip phenomenon [26]. At this time, a large amount of material will be spalling off on the surface of the pure TPU sample, tearing and pits of the material will occur on the surface of the sample, and a large number of abrasive particles will exist in the lubricating medium, which will be discussed in the following. During the friction process, the contact temperature of TPU composites will rise due to frictional heating, and this temperature rise may affect its tribological properties because the thermal resistance of TPU is limited [27]. Moreover, the wear behavior of high-performance polymers at different temperatures shows that temperature changes will affect the brittle-ductility transition behavior of materials [28] and thus affect their wear properties. For BF samples, the basalt fibers were completely pulled out of the matrix by extreme shear force, and many structural defects appeared on the surface of the samples. When subjected to great pressure, UHMWPE fiber has a highly linear molecular chain structure and thus has good self-lubricating properties [29]. At the same time, bamboo fiber also contains natural vegetable lubrication components [30], so UF and BMF show better tribological properties. Under high load, the TPU on the surface of UHMWPE fiber is worn off, and the UHMWPE fiber with better wear resistance is exposed, but it is not pulled out, and the tribological properties of the overall structure are improved. Under the test condition of 0.7 MPa, the UF sample shows the best performance. In addition, for most samples under the same pressure, the average friction coefficient will decrease with the increase in rotational speed, which may be because when the rotational speed increases, the flow rate of distilled water between the two interfaces will accelerate, the lubrication pressure between the two interfaces will decrease, and the internal and external lubrication pressure difference will appear. Under the action of external liquid pressure, more distilled water enters the gap [31], the water film thickness increases, the lubrication state becomes better, and the friction coefficient decreases.

Table 5 shows the real-time changes in friction coefficients of four materials under six different working conditions, which can more directly observe the changes in friction coefficients of materials over time and under different working conditions. The overall value comparison is similar to the average friction coefficient in this section. It is worth noting that the real-time friction coefficient of polymer materials under high-load conditions is more stable than that under lower load conditions, especially when fiber is added as the reinforcement phase of the sample. This may be because the high load will make the friction pair fit more tightly, and it is easier to produce fiber fragments and friction films [32], and the lubrication conditions are improved. As can be seen from Table 5, when the load increases from 0.5 MPa to 0.7 MPa, the overall friction coefficient of TPU and BF samples increases, while that of UF and BMF samples decreases, which is consistent with the previous explanation. At the same time, under the condition of high load (0.7 MPa), the friction coefficients of the four materials are significantly compared. The real-time friction coefficient of the TPU sample remained at the highest level and fluctuated the most. The friction coefficient curve of TPU may fluctuate greatly because a large number of materials on the surface are stripped, the surface is pitted, a large number of abrasive particles exist in the lubricating water, and the friction process is extremely unstable. However, the friction coefficient of UF sample is the smallest among the four materials most of the time, showing a trend of continuous decline during the test. One of the most important reasons is that after TPU is worn off by the tin bronze plate, UHMWPE fiber, which has better wear resistance than TPU [33], plays a major role, and the friction coefficient continues to decrease. The friction coefficients of UF and BMF samples are maintained at a good level and are accompanied by a slow decline trend, which can also prove that UHMWPE fiber and bamboo fiber have better self-lubrication ability under high load, thus improving the lubrication environment. Occasionally, the friction coefficient of BMF samples can be found to fluctuate slightly, which indicates that the mechanical properties of bamboo fiber need to be improved. It should also be noted that by observing the friction coefficient curve of BF sample under 0.7 MPa test conditions, it can be found that after 7200 s of friction testing, the friction coefficient of BF does not decrease but will continue to rise most of the time. Combining the surface microstructure and wear mechanism of the sample, we can find that the basalt fiber in the BF sample has poor compatibility with the TPU matrix. As a result, many fibers are completely pulled out of the matrix, and the friction interface conditions are very bad.

### 3.4. Wear Mass Loss Analysis

**Figure 5:**
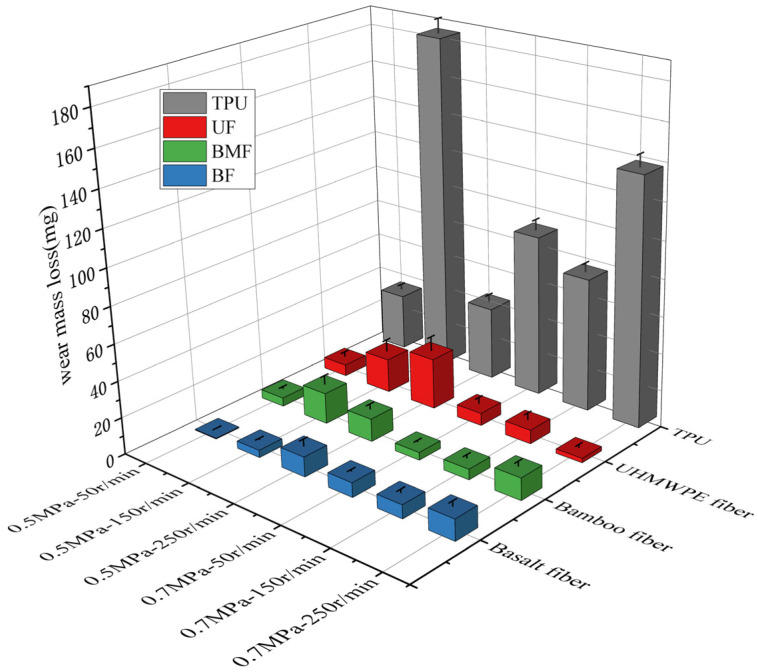
Wear mass loss of samples under different test conditions.

The wear mass loss of materials is also one of the important indexes to measure the tribological properties of polymers. Figure 5 shows the wear mass loss of the samples under different test conditions. It can be found that the wear mass loss of the TPU composite material is significantly reduced after the addition of three kinds of staple fibers, which indicates that the addition of three kinds of short fibers has a positive impact on the wear resistance of TPU material.

**Figure 6:**
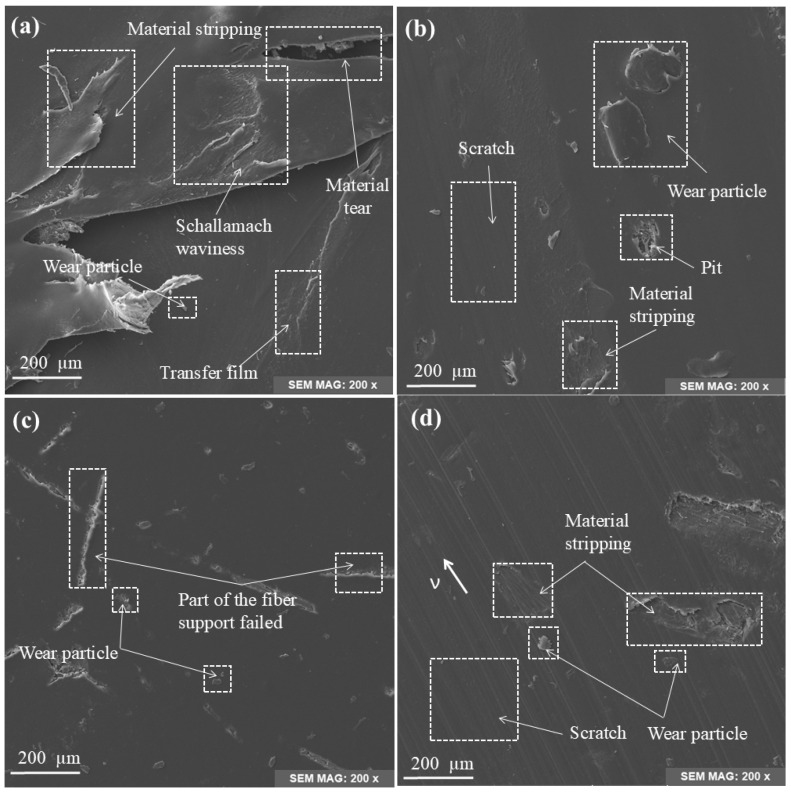
Surface microwear morphology of TPU-matrix composites: (**a**) TPU, (**b**) UF, (**c**) BF, and (**d**) BMF.

**Figure 7:**
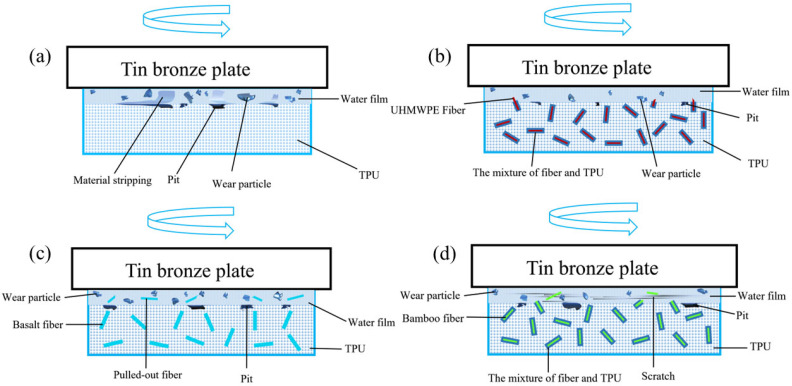
Wear mechanisms: (**a**) TPU, (**b**) UF, (**c**) BF, and (**d**) BMF.

It is worth noting that in all friction tests, the wear quality of TPU samples is the largest, which indicates that the addition of fibers has a positive effect on the wear resistance of TPU composites. By observing the surface of the sample and analyzing the wear mechanism (Figure 6 and Figure 7), we can find that a large number of materials on the surface of pure TPU are spalling, which inevitably makes the wear quality extremely high. In addition, the accumulation of friction charges may increase friction and wear. The mechanical action on the contact surface can lead to the formation of free radicals and electron transfer, resulting in cationic and anionic polymer fragments, forming charge accumulation [34], and this charge distribution will increase the electrostatic interaction between the surfaces, thereby increasing the coefficient of friction and aggravating the wear of the material [35]. The minimum wear of the basalt fiber-reinforced composite was only 0.4mg, which is 98.7% lower than the pure TPU. This may be because the high hardness and strength of the basalt fiber enable it to effectively share the load during the friction process while improving the fatigue resistance of the material, reducing the microscopic crack growth during the friction process, and thus reducing the wear of the TPU matrix material [36]. At the same time, at 0.5 MPa, the fluctuation amplitude of BF wear mass is the smallest when the load and speed change, and the influence of test conditions on the wear quality is the least. This may be because basalt fibers enhance the mechanical strength and fatigue resistance of TPU materials. However, under high-load conditions, UF samples performed best, followed by BMF samples, and BF samples did not perform as well as when subjected to relatively low loads. This is because in the high-stress field, the basalt fibers are uprooted from the matrix by extreme shear forces, and the abrasive particles increase along with the deteriorating lubrication conditions. However, UHMWPE fiber has better compatibility with the matrix; only the TPU and a small amount of fiber body are worn off, and the wear mass loss is minimal in general. The BMF sample is between BF and UF.

### 3.5. Wear Surface Topography of the Specimens

In order to better evaluate the tribological properties of four kinds of short fiber-reinforced composites and more accurately describe the friction and wear of the materials, scanning electron microscopy (SEM) was used to observe the surface of the tested composites. According to previous studies, the actual maximum wear of water-lubricated bearings occurs at low-speed and heavy-load conditions, so SEM analysis mainly focuses on the morphology of samples tested at 0.7 MPa pressure [37].

Figure 6 shows the surface microstructure of TPU-based composites. On the whole, the surface wear condition of the three FRC samples is better than that of the pure TPU sample, which indicates that the three reinforced fibers can effectively improve the tribological properties of TPU materials. It can be observed from Figure 6a that the surface of TPU produces a thermal effect under the condition of low speed and heavy load, resulting in deformation of the TPU surface and thus affecting the sticking-slip phenomenon. For transfer film, large pieces of material spalling and tearing are generated on the surface of the sample, accompanied by a large amount of wear chips. At the same time, the Schallamach waves are caused by the periodic ripple deformation caused by the adhesive force and shear force on the contact surface, which indicates the existence of adhesive wear and abrasive wear, and the friction environment of the specimen surface is poor, and the friction mass loss is great. By observing Figure 6b–d, it can be found that there are slight scratches and pits on the UF surface, there are wear particles on the surface, and the surface roughness increases significantly, showing the characteristics of abrasive wear. At the same time, part of the fiber connected to the matrix can be observed from the pits, which indicates that the TPU near the fiber has been worn off, while a part of the wear-resistant UHMWPE fiber is still left in the matrix, as shown in Figure 7b. The BF surface has a small amount of abrasive particles and more fiber support failures, which are signs of high-stress areas during friction, indicating that the material has significant plastic deformation and breakage during friction. Combined with the previous analysis of BF friction coefficient and wear mass loss, we can see that these linear grooves indicate that the basalt fibers have been completely pulled out of the matrix, and the structural integrity of the sample is insufficient. The deep scratches present on the BMF surface extend along the direction of friction, showing the action of high stress and shear forces. In addition, there are tiny cracks and particle residue on the surface, and a small number of fiber bundles can be seen in the cracks, indicating that the base material and some fibers have been worn and produced fragments.

### 3.6. Wear Morphologies of Tin Bronze Plates

**Figure 8:**
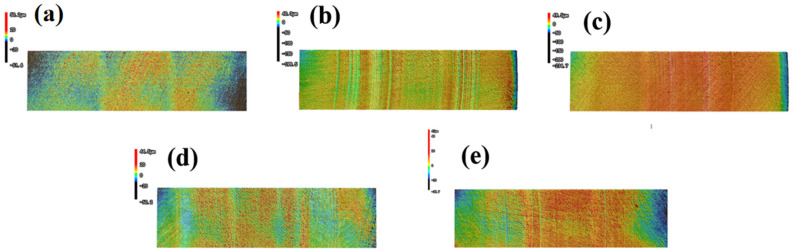
Wear morphologies of the tin bronze plates at 0.7 MPa contact specimens: (**a**) untested, (**b**) UF, (**c**) TPU, (**d**) BF, and (**e**) BMF.

**Figure 9:**
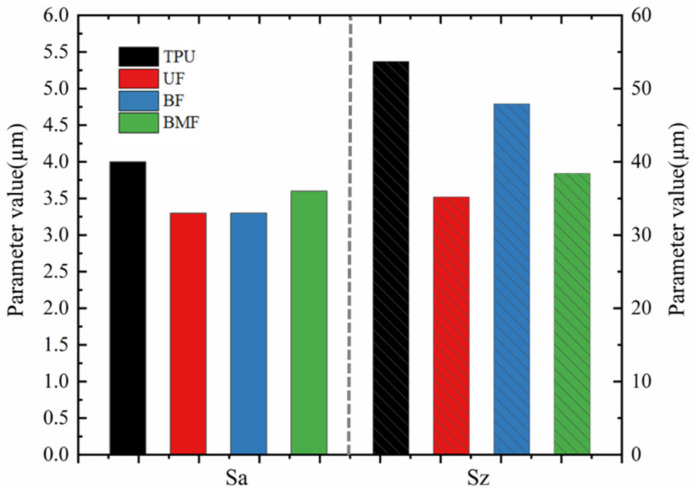
Surface characteristic parameters of tin bronze plate.

The friction pair of the water-lubricated bearing in this test is composed of a metal drive shaft and a polymer bearing [38]. Due to the complexity of the preparation process and the fine size requirements, the drive shaft is much more expensive than the bearing. Therefore, the wear morphology of the tin bronze plate used in the test is compared and analyzed in this paper. As with Section 3.5, considering that the greatest wear may occur under the highest load, tin bronze plates at 0.7 MPa were selected for analysis. Figure 8 and Figure 9 are the wear surface topography of the tin bronze plate and the comparison of selected characteristic parameters, respectively.

It can be seen from the surface morphology that the surface of the untested tin bronze plate is relatively flat, and there are only uniform marks caused by the killing in the polishing process. Other tin bronze plates tested all show abrasion marks of varying shades. Compared with the surface of the tin bronze plate that has not been tested, the roughness peak height difference of the tin bronze plate grinded with pure TPU is the most obvious, and there are a few scratches and grooves on the surface of the tin bronze plate grinded with BF. However, due to the high hardness of BF itself, it can be observed that the surface of the tin bronze plate presents a large number of microplastic convex structures [39], but it remains relatively flat on the macro level. The surface of the tin bronze plate milled with UF and BMF showed more uniform scratches and adhesion, and it was also observed that part of the wear chips and adhesion filled the cracks of the scratches, improving the surface smoothness and friction properties. In Figure 9, Sa represents the average roughness, Sz represents the maximum peak and valley height, and both the average roughness and maximum peak and valley height of TPU remain at the highest level. Based on the above analysis, it may be because the deformed TPU is attached to the surface of the copper disk. The test results show that all three staple fibers can improve the friction environment of the materials. The surface roughness of the tin bronze plate ground with UF and BF is the smallest, and the surface peak of the tin bronze plate ground with UF and BMF is relatively gentle. The test results are consistent with the surface images of the samples taken by SEM. The wear debris and stick-slip TPU on the surface of the UF and BMF samples are embedded in the surface of the tin bronze plate, which has a positive influence on its roughness and surface peak. At the same time, the depression caused by the failure of many fibers to support the BF sample surface may also lead to sharp surface peaks.

## 4. Conclusions

In this study, the properties of thermoplastic polyurethane (TPU)-based composites were investigated under conditions similar to those of water-lubricated stern tube plain bearings. Three short fibers were added to TPU, and the tribological behavior of these three composites was studied under different loads and rotational speeds. Through the comprehensive analysis of mechanical properties, friction coefficient, wear mass loss, and surface topography, the conclusions drawn from this investigation are shown as follows:

1. In this paper, BF shows excellent mechanical properties, and the hardness and tensile strength are relatively the largest among the four materials. With the addition of UHMWPE fiber, the hardness of TPU will decrease, but the elongation at break will increase significantly, which indicates that UF is very flexible and can produce huge deformation. In addition, the water swelling of the three fiber-reinforced composites (FRCs) meets military standards, with BF providing the best performance.
2. The different speeds and loads applied in the test will affect the friction and wear properties of the composites. Under boundary lubrication, the frictional properties of TPU matrix composites are affected by contact mechanics and viscoelasticity. TPU increases friction and wear, but reinforced fillers improve wear resistance. The friction coefficient of BF was only 0.088 under the working conditions of 0.5 MPa and 250 r/min, which was 70.57% lower than that of pure TPU material. And in all the tests, the minimum wear of the BF is only 0.4 mg, which is also the smallest of all the materials under all conditions, and a decrease of 98.69% compared to TPU. However, under the test conditions of high load and low speed, UF samples showed lower friction coefficients and better wear resistance, which may be related to the good compatibility between UHMWPE fiber and TPU. The increase in sliding speed helps to form a water lubrication film, while the pure TPU surface appears to have a large number of material spalling and a large number of abrasive particles in the lubricating water, resulting in high wear and friction fluctuations, and the surface produces Schallamach waves.
3. Under the normal load (0.5 MPa), the superior mechanical properties of basalt fiber, such as high hardness, bear most of the load of the BF sample, reducing the contact area and improving the friction performance. However, under high load (0.7 MPa), the basalt fiber support partially fails, resulting in obvious stress concentration, plastic deformation, and fracture, and the whole fiber is pulled out from the matrix, destroying the structural integrity of the material. The self-lubricity of the relatively soft UHMWPE fiber and bamboo fiber plays a major role, and their compatibility with the TPU matrix is relatively better. The fiber is fixed in the matrix and hardly pulled out. At the same time, part of the grinding chips are filled into the copper tray groove, which improves the surface finish, and the friction properties of UF and BMF are also improved.
4. This study proves that three different short fibers can improve the tribological properties of the TPU matrix in different degrees, which provides an experimental basis for improving the tribological properties of environmentally friendly water-lubricated bearing composites. It is necessary to further study the binding force between the reinforced fiber and the matrix in order to further improve the comprehensive properties of the material.

## Figures and Tables

**Table 1:** Qsn7-0.2 tin bronze disc element composition.

Element	Cu	Zn	Sn	Ni	Al	Pb	Others
Proportion	90–92%	0.3%	6–8%	0.2%	0.01%	0.02%	0.15%

**Table 2:** Properties of TPU.

	Density (g/cm ^3^)	Tensile Strength (MPa)	Elongation at Break (%)	Poisson Ratio	Hardness (Shore D)
TPU(S172DL)	1.23	34.1 (23 °C)	274 (23 °C)	0.38	72

**Table 3:** Qsn7-0.2 Mechanical properties of tin bronze plates.

	Material	Yield Strength (MPa)	Tensile Strength (MPa)	Hardness (HB)
Tin bronze plates	Qsn7–0.2	≥170	≥355	≥70

**Table 4:** The average volume changes in samples for different soaking times.

Sample	TPU	UF	BF	BMF
Volume growth after 12 h	0.303%	0.301%	0.210%	0.405%
Volume growth after 24 h	0.548%	0.547%	0.318%	0.504%
Volume growth after 7 d	0.576%	0.551%	0.403%	0.950%

**Table 5:** Real-time friction coefficients under different test conditions.

	Load	0.5 MPa	0.7 MPa
**Rotational** **Speed**			
50 r/min		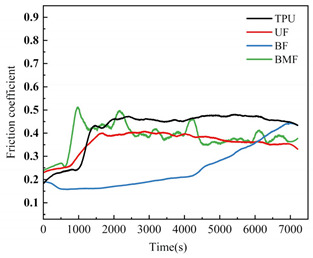	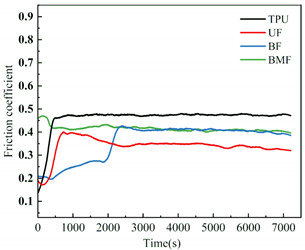
150 r/min		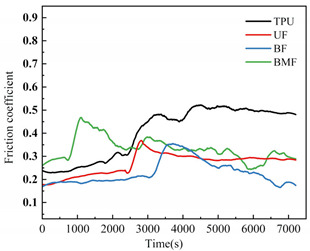	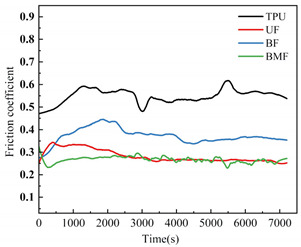
250 r/min		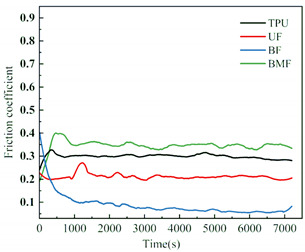	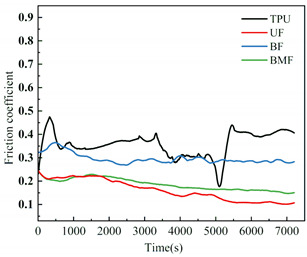

## Data Availability

The data presented in this study are available on request from the corresponding author.
